# Swarm shedding in networks of self-propelled agents

**DOI:** 10.1038/s41598-021-92748-1

**Published:** 2021-06-29

**Authors:** Jason Hindes, Victoria Edwards, Klimka Szwaykowska Kasraie, George Stantchev, Ira B. Schwartz

**Affiliations:** 1 U.S. Naval Research Laboratory, Washington, DC 20375 USA; 2 University of Pennsylvania, Philadelphia, PA 19104 USA; 3 Georgia Tech Research Institute, Atlanta, GA 30318 USA

**Keywords:** Computational biophysics, Biological physics, Complex networks, Nonlinear phenomena, Phase transitions and critical phenomena, Applied mathematics, Mechanical engineering

## Abstract

Understanding swarm pattern formation is of great interest because it occurs naturally in many physical and biological systems, and has artificial applications in robotics. In both natural and engineered swarms, agent communication is typically local and sparse. This is because, over a limited sensing or communication range, the number of interactions an agent has is much smaller than the total possible number. A central question for self-organizing swarms interacting through sparse networks is whether or not collective motion states can emerge where all agents have coherent and stable dynamics. In this work we introduce the phenomenon of swarm *shedding* in which weakly-connected agents are ejected from stable milling patterns in self-propelled swarming networks with finite-range interactions. We show that swarm shedding can be localized around a few agents, or delocalized, and entail a simultaneous ejection of all agents in a network. Despite the complexity of milling motion in complex networks, we successfully build mean-field theory that accurately predicts both milling state dynamics and shedding transitions. The latter are described in terms of saddle-node bifurcations that depend on the range of communication, the inter-agent interaction strength, and the network topology.

## Introduction

Much attention has been given to the study of multi-agent swarms that can self organize and form complex spatiotemporal patterns from very basic rules governing individual dynamics^1–3^. Natural swarms typically involve the coupling of large numbers of mobile agents, and can be seen in many fascinating biological systems from flocks of birds^4–6^, to schools of fish^7,8^, crowds of people^9^, and colonies of bacteria and insects^10,11^. Similar self-organization phenomena occur in self-propelled, active-matter systems^12–16^. Both theoretical and applied research has focused on understanding the principles underlying collective motion^1,2,17–24^, and how such principles can be instantiated in mobile-robotic systems^25–29^. Applications for the latter range from mapping^30^, to exploration^27^, and resource allocation^31–33^.

Agent interactions in both natural and decentralized robotic swarms are typically sparse and local due to finite bandwidth and communication range^29,34,35^. Sparse and heterogeneous network effects on swarming are understood analytically, mostly within the context of controlling teams of mobile agents through decentralized, average consensus algorithms^20,36–38^. Typically, such systems involve sharing speed and heading data directly among agents, and are somewhat limited in their dynamics, e.g., to flocking, where consensus forms around a network-wide velocity. On the other hand, physically inspired models, where collective motion emerges from the more basic interplay of position-dependent forces and self-propulsion energy, have typically assumed global, homogeneous, or lattice communication topology^39–45^. For instance, early robotics experiments based on such nonlinear-physics models, also assumed all-to-all coupling^42,46^—making them difficult to scale to larger systems and less controlled environments. Since the latter class of models derive from basic physical principles, they showcase a broader spectrum of emergent motion patterns, and can more easily incorporate, e.g., active-matter dynamics^15,43^ and collective motion on arbitrary surfaces^47^. Recent work has begun to address network structure in such physically-inspired swarming systems, including how topology affects robustness to noise^48^ and how heterogenous topology drives the formation of hybrid motion states^49^. Yet, much remains unknown about how complex topology influences the dynamical stability of swarms with general nonlinear interactions and under what circumstances a sparse swarming network can maintain coherent motion of all its agents—especially in the much broader range of collective-motion patterns without rigid velocity consensus.

To make progress, we consider a well known physics-based model of mobile agents moving under the influence of self-propulsion, damping, and pairwise interaction forces^41,43,48,50^, to which we add explicit sparse networks that mediate and constrain the inter-agent interactions^42,49^. In the absence of interactions, each swarmer will tend to a fixed speed, which balances its self-propulsion and damping but has no preferred direction^47^. The agents are assumed to interact through a network, whose topology is *fixed in time*, and given by a *simple static graph* with an adjacency matrix: $A_{ij} = 1$, if agents *i* and *j* are connected, and zero otherwise^51^. The matrix $A_{ij}$ will be generated from a variety of standard graph models specified below. In addition to the topology, the interaction between two agents will be associated with a strength that is assumed to decay exponentially with their relative distance, namely $\exp \{-|{\mathbf{r}}_{j}-{\mathbf{r}}_{i}|/l_{2}\}$, where ${\mathbf{r}}_{i}$ is the position-vector for the *i*th agent in three spatial dimensions (and similarly for agent *j*), and $l_{2}$ is a constant measuring the characteristic length scale for maintaining connections. The connection strength can be thought of as an edge weight in the corresponding (weighted) interaction graph model, which for example is commonly used as a simple approximation for ad-hoc wireless networks^52^. Altogether, the interaction graph matrix takes the form

1 $$\begin{aligned} W_{ij} =A_{ij}\exp \{-|{\mathbf{r}}_{j}-{\mathbf{r}}_{i}|/l_{2}\} \end{aligned}$$

for agents *i* and *j*.

Given an interaction graph, we assume that there is a force between two agents, either a real physical force or a control force, that tends to minimize a scalar potential function of the agent positions. As in many other works, we assume that the interaction force is elastic (spring-like) and tends to maintain a characteristic separation between two agents, $l_{1}$^41,43,48,50,53^. Combining all of the basic physics-ingredients gives a dynamic model for the *i*th agent

2 $$\begin{aligned} \ddot{{\mathbf{r}}}_{i}=\left[ \alpha -\beta |\dot{{\mathbf{r}}}_{i}|^{2}\right] \dot{{\mathbf{r}}}_{i}\;\;\;\;+ \lambda \sum _{j}A_{ij}\;e^{\frac{-|{\mathbf{r}}_{j}-{\mathbf{r}}_{i}|}{l_{2}}}\frac{{\mathbf{r}}_{j}-{\mathbf{r}}_{i}}{|{\mathbf{r}}_{j}-{\mathbf{r}}_{i}|}\left( |{\mathbf{r}}_{j}-{\mathbf{r}}_{i}|-l_{1}\right) , \end{aligned}$$

where $\alpha$ is a self-propulsion constant, $\beta$ is a damping constant, and $\lambda$ is a coupling constant^39–41,50,54^; the symbol $\ddot{{\mathbf{r}}}_{i}$ denotes the acceleration of the *i*th agent, while $\dot{{\mathbf{r}}}_{i}$ denotes its velocity. Note that the aforementioned weighted interaction matrix, $W_{ij} = A_{ij}\exp \{-|{\mathbf{r}}_{j}-{\mathbf{r}}_{i}|/l_{2}\}$, that appears in the second term of Eq. (2) controls directly the dynamics of pairwise interactions among swarming agents, and in that sense it serves as a surrogate for an actual communication model. This simplification allows us to build intuition about the behavior of a swarming system with heterogeneous sparse connectivity while avoiding the complexity of modeling communication channel effects and wireless network implementations.

Our approach in the following is to study the stability of basic swarming patterns in the model Eq. (2), and in particular understand how pattern stability is lost in a given network, $A_{ij}$, as we change the communication range $l_{2}$, and the strength of inter-agent coupling, $\lambda$. A successful theory in this regard should predict how strong the coupling must be, and how far the communication range, in order to stabilize collective motion states in given a network. Such a theory could also provide insights for guiding robotics experiments with autonomous ground, surface, and aerial vehicles^42,53,46^, which have used Eqs. (2), and similar variants, as an underlying control law. In particular, these experiments were effectively all-to-all in terms of communication, and thus, our analysis can help scale-up similar experiments to larger robotic swarms in less controlled environments.

## Methods

**Figure 1:**
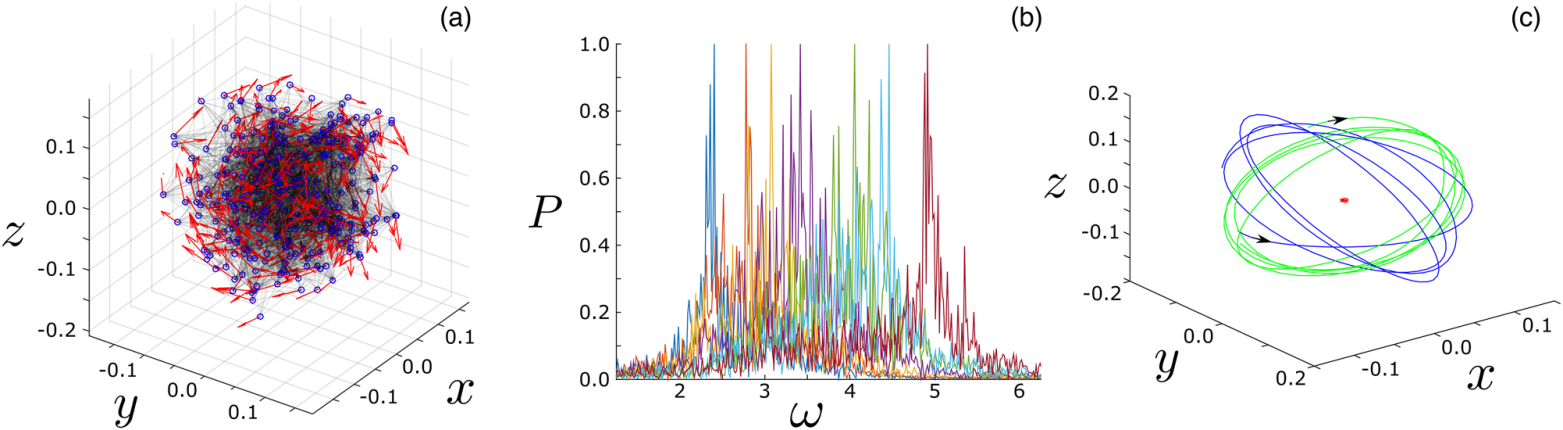
Milling state in self-propelled swarming networks. (**a**) Snap-shot of milling in which agents are drawn with blue circles, velocities with red arrows, and network edges with black-lines. (**b**) The Fourier spectra (normalized, absolute value) of seven randomly selected agents drawn with different colors. For reference, the leftmost spectrum corresponds to a $k = 8$ agent while the rightmost corresponds to $k = 40$. (**c**) Trajectory of a $k = 10$ agent (blue), $k = 20$ agent (green), and the swarm center of mass (red). The network topology was a Waxman geometric random graph with 300 agents and an average degree of 20. Other model parameters are: $\alpha = 1$, $\beta = 5$, $l_{1} = 0.1$, $l_{2} = 1$, and $\lambda = 2$.

First, when simulating the model Eq. (2) with random initial positions and velocities we find two primary collective-motion patterns: flocking and milling^55^. In the former, a swarm’s center of mass translates at a steady, fixed velocity. Agents move on average with the center-of-mass velocity and undergo slow oscillations around a fixed relative formation that is a steady-state solution of the over-damped dynamics^56^. In contrast, in the milling state (MS) agents rotate around a stationary center of mass with no macroscopic consensus in velocity. Whether or not a swarm converges to one of these two states (or possibly others), depends on initial conditions, swarm parameters, and network topology. In this work, we focus on the MS since it emerges from the broadest range of initial conditions, e.g., random initial headings for the agents. An example MS is shown in Fig. 1 given a Waxman random geometric graph of connections for the topology $A_{ij}$, where nodes are connected according to an exponential probability distribution in their initial separations^57^. The network had a distribution of connections per node (or degree, *k*) that was somewhat heterogeneous, ranging from $k = 4$ to $k = 42$, with an average and standard deviation, $\langle k \rangle = 20$ and $\sigma =7 \;$, respectively; see supplementary material appendix for more network details. In terms of spatio-temporal initial conditions, throughout this work, we assign every agent a location selected uniformly at random inside a cube with unit side length, centered at the origin. Similarly, the velocities are assigned uniformly at random inside a velocity-cube with unit side length, centered at zero-velocity. Then, Eq. (2) is integrated to a time $t = 1000$, so that the resulting behavior is non-transient. We find that such conditions are sufficient to produce stable milling patterns.

Important features of the MS dynamics can be seen in both panels (a) and (b) of Fig. 1. In the first, a snap-shot in time shows that agents are arranged at various instantaneous distances from the center of the swarm with a broad distribution of velocities (there is no easily discernible pattern in the heading arrows). Qualitatively, higher-degree agents wander near the center, while lower-degree agents wander at the periphery. In the second panel (b), we plot the normalized Fourier spectra for several example agents, where the peak frequency is set to unity. Though the Fourier spectra are non-trivial and broad in general, a basic pattern emerges when we note that the peaks follow the ordering of degree, from left to right: low to high degree nodes.

**Figure 2:**
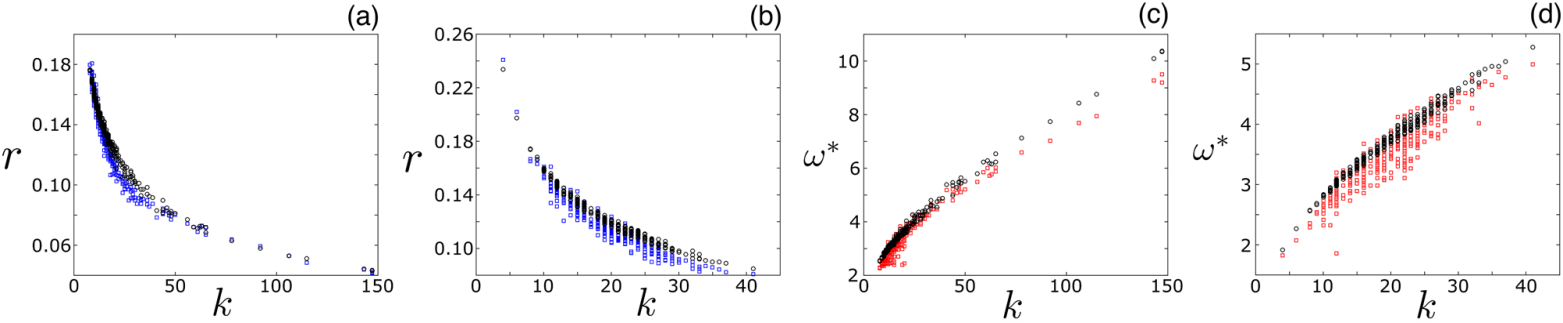
Averaged milling state oscillations. The average distance to the swarm’s center of mass versus the agent degree for a power-law network (**a**) and a Waxman geometric random graph (**b**). Simulation results are shown with blue squares and mean-field predictions with black circles from solving Eqs. (3a–3b). Peak frequency from the Fourier spectra versus agent degree for the same power-law network (**c**) and Waxman geometric random graph (**d**). Simulation results are shown with red squares and mean-field predictions with black circles. Each network was composed of 300 agents with an average degree of 20. Other model parameters are identical to Fig. 1.

These observations can be made more precise by plotting the time-averaged distance to the swarm center of mass and the peak frequency versus the degree. Both are computed by integrating Eq. (2) over an additional $\Delta t = 200$ from the initial conditions specified in the first paragraph of ‘Methods’ section. Examples are shown in Fig. 2 for two network topologies $(A_{ij})$: a power-law degree distributed network and a Waxman graph. The power-law network was constructed using the configuration model and a fraction of nodes with degree *k*, $g(k) \sim k^{-2.5}\;$^58^. The network was also heterogeneous with an average degree $\langle k \rangle = 20$ and a standard deviation $\sigma =20 \;$; further network details are given in the supplementary material. Simulation results are shown with blue and red squares. Despite the instantaneous complexity of the MS dynamics illustrated in Fig. 1, Fig. 2 suggests that the approximate behavior of every agent is to rotate on an orbit with some steady-state distance to the swarm’s center at a frequency that depends on its location in the network (and particular, on its topological degree *k*). To further illustrate the rotational dynamics, we show three example trajectories for a $k = 10$ agent (blue), a $k = 20$ agent (green), and the swarm center of mass (red) in Fig. 1c. The next step is to predict the center-of-mass distances *r* and frequencies $\omega$, and understand their dependence on the model parameters and topology in quantitative detail.

### Milling state mean field

We can approximate the behavior plotted in Fig. 2 by building a steady-state mean-field description of the MS. First, let us adopt spherical coordinates for convenience, e.g., for node i, ${\mathbf{r}}_{i} = r_{i}\cos {\phi _{i}}\sin {\theta _{i}}\;\hat{{\mathbf{x}}}+r_{i}\sin {\phi _{i}}\sin {\theta _{i}}\;\hat{{\mathbf{y}}}+r_{i}\cos {\theta _{i}}\;\hat{{\mathbf{z}}}$. Second, let us assume that every node travels on a circular orbit (approximately) with fixed radius and frequency, i.e., $r_{i}$ and $\omega _{i}$, respectively for node *i*. Third, let us orient our axes such that a test node *i* oscillates with $\theta _{i}=\omega _{i}\;t$ and $\phi _{i} = 0$. Fourth, let us substitute the ansatz, ${\mathbf{r}}_{i} = r_{i}\sin {\omega _{i}\;t}\;\hat{{\mathbf{x}}}+r_{i}\cos {\omega _{i}\;t}\;\hat{{\mathbf{z}}}$, for node *i* into Eq. (2). The result is three equations for each node that must be satisfied (one for each component of acceleration).

The final mean-field step is to approximate the interaction-sums in Eq. (2), by assuming that there is *no correlation* between the angles $\theta _{i}$, $\theta _{j}$, $\phi _{i}$, and $\phi _{j}$ in the MS for any connected nodes *i* and *j*—the only correlation is in the radii by assumption. Therefore, from the perspective of node *i*, the angles for node *j* in the MS are equally-likely to take on any values during its orbit, and thus for every interaction-term in Eq. (2) we multiply by the probability that the angles are found within a small interval centered around $\theta _{j}$ and $\phi _{j}$, $\sin {\theta _{j}}d\theta _{j}d\phi _{j}\big /4\pi$ and integrate over $\theta _{j}$ and $\phi _{j}$. Repeating the above for every node results in *N* root equations, e.g., $F_{i}(r_{1},r_{2},\ldots ,r_{N}) = 0$ for node *i*, where

3a $$\begin{aligned}F_{i}=\;\frac{\alpha }{\lambda \beta r_{i}}\;+\;\sum _{j}A_{ij} \int _{0}^{\pi } \frac{\sin {\theta _{j}d\theta _{j}}}{2}(r_{j}\cos {\theta _{j}}-r_{i})\;e^{-d_{ij}/l_{2}}\left( 1-\frac{l_{1}}{d_{ij}} \right) \;, \end{aligned}$$

3b $$\begin{aligned}d_{ij}(r_{i},r_{j},\theta _{j})=\sqrt{r_{j}^{2}+r_{i}^{2}-2r_{i}r_{j}\cos {\theta _{j}}}, \end{aligned}$$

and

4 $$\begin{aligned} \omega _{i}=\;\sqrt{\alpha /\beta }/r_{i} \end{aligned}$$

The Eqs. (3a–3b) can be solved by numerically integrating the polar-angle $\theta$-integrals and computing the *r*’s through a quasi-Newton evaluation of Eqs. (3a). We note that in the limit of zero-repulsion, $l_{1} \rightarrow 0$, each integral can be solved in closed-form and written as explicit functions of $r_{i}$ and $r_{j}$. Numerical solutions of Eqs. (3a–3b) are plotted in Fig. 2 alongside simulation values and show good agreement, despite the very different network topologies used in each example, and thus demonstrating the robustness of our mean-field predictions to topological variation.

It is important to note that the behavior implied by Eq. (3a) is a mean-field approximation to the actual swarming dynamics in the MS. To get a better sense of this we return to Fig. 1c, which shows sample trajectories over a time interval of $\Delta t = 10$ for a $k = 10$ agent (blue), a $k = 20$ agent (green), and the swarm center of mass (red). For the first two agents, we can see that instead of rotating around a fixed plane, in fact, each agent wobbles and precesses over the course of several periods of its oscillation. However, as long as the precession occurs slowly relative to the dominant frequency (as they do in these examples) the mean-field approximation is fairly accurate. Another assumption of the mean field is that the swarm center of mass is stationary in time. In actuality, the center of mass fluctuates with a standard-deviation that is *O*(1/*N*). Note that in Fig. 1c the red trajectory represents a small, finite-size vibration compared to the large amplitude oscillations of individual agents.

Notably, in the limit of long-range communication, $l_{2} \gg 1$, and weak repulsive force between agents, $l_{1} \ll 1$, solutions approach

5 $$\begin{aligned} r_{i}\approx \sqrt{\alpha \Big / \beta \lambda k_{i}} \end{aligned}$$

and $\omega _{i} \approx \sqrt{\lambda k_{i}}$ , implying that the MS radii are expected to scale inversely with the square root of the coupling ,$\lambda$, and the degree of agents^42^. Note the degree of node *i*, $k_{i} = \sum _{j}A_{ij}$.

## Results

**Figure 3:**
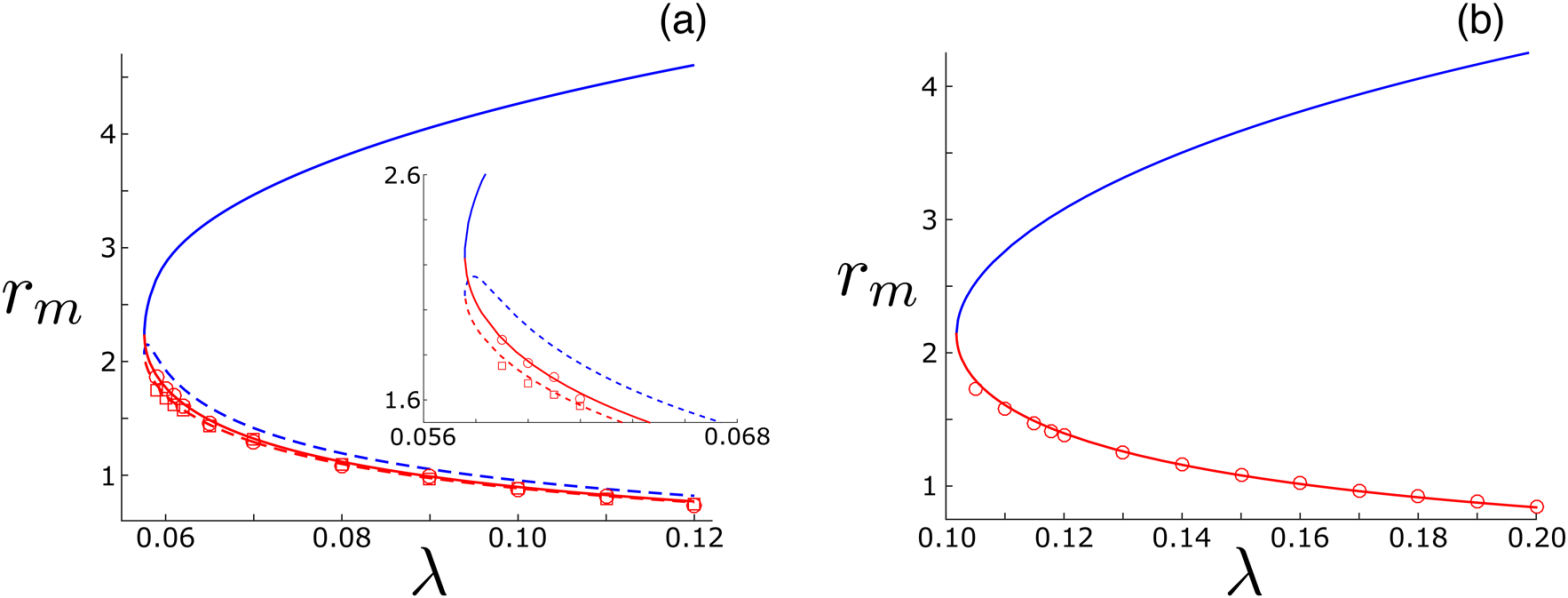
Distance from a swarm’s center versus coupling for agents with the lowest degree in their network. (**a**) Power-law network where simulation-determined values are plotted with red circles and squares for two agents that are directly connected and ejected at the first shedding transition. Mean-field predictions from solving Eqs.(3a–3b) are drawn for each node with solid and dashed lines: stable (red) and unstable (blue). The inlet panel shows the same plot zoomed-in to the bifurcation. (**b**) Similar plot for a Waxman network topology, where only one agent is shed. Other model parameters are identical to Fig. 1.

In the MS a constant-magnitude centripetal force is supplied to every agent by the sum-total of its network interactions, which are weighted by the coupling constant, $\lambda$. The corresponding centripetal acceleration is constrained by the fact that the self-propulsion and damping forces must also balance, and hence the speed of every agent is $\sqrt{\alpha /\beta }$ within the mean-field approximation. Since, we are interested in the effect of reducing the coupling between agents in the network, consider what happens when $\lambda$ is reduced, for example. As $\lambda$ decreases, the average distance from the swarm’s center increases, according to Eq. (5), and agents are less tightly held by the interaction force. Two examples of this trend are shown in Fig. 3, where radii of the lowest-degree agents in two networks are plotted in red as function of $\lambda$. At some critical point $\lambda _{c}$, the lowest-degree agents approach the communication length scale, $r \sim l_{2}$, and it becomes stable for the network to simply eject such agents from the MS, and zero-out their interactions by letting them fly off to infinity: $W_{ij} \rightarrow 0$ for ejected node *i*. We call this transition in general, swarm *shedding*.

Qualitatively, if the low-degree agents are connected to higher-degree agents, as is typically the case in random degree-heterogeneous networks like the Waxman and power-law networks, shedding will only involve isolated lowest-degree agents. Both examples in Fig. 3 illustrate this pattern. In such networks, shedding occurs in a distinct sequence: first the lowest-degree agent is shed at some $\lambda _{c,1}$ , then the second lowest at some $\lambda _{c,2} < \lambda _{c,1}$ , etc. We denote these transitions as *localized shedding* from the MS, since instability is associated with one (or ${\mathscr {O}}(1)$ ) agents. On the other hand if the lowest-degree agents are connected to other low-degree agents, as in weakly connected homogenous networks where most agents have $k \sim \langle k \rangle \sim {\mathscr {O}}(1)$, then when a low degree agent is shed it will cause other agents to effectively go out of communication range and be shed, resulting in a cascade of low-degree agent shedding. Because instability involves ${\mathscr {O}}(N)$ agents at the critical point in this case, we call such transitions *delocalized shedding*.

**Figure 4:**
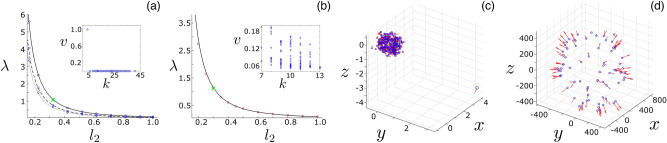
Shedding of agents from milling states. (**a**) Critical coupling for localized shedding in a Waxman geometric random graph. Points indicate simulation-determined values while lines indicate mean-field predictions. Plotted are the first three shedding transitions: $k = 4$ (circles, solid line), $k = 6$ (squares, dashed line), and $k = 8$ (diamonds, dotted line). (**b**) Critical coupling for the single, delocalized shedding transition in a Watts–Strogatz random graph. The Watts–Strogatz graph was composed of 100 agents with an average degree of 10. Inlet panels in (**a**) and (**b**) show the predicted unstable mode associated with the upper mean-field bifurcation by solving Eq. (6). Example snapshots for $\lambda$ just below the respective transitions are shown in (**c**) and (**d**) for the two graphs. Locations for the snapshots within the diagrams (**a**) and (**b**) are specified with a green x. Other model parameters and plotting conventions are identical to Fig. 1.

Examples of each kind of shedding are shown in Fig. 4. On the left, a sequence of localized sheddings occurs in a swarm with a Waxman network topology. In subpanel (a) we plot the first three transitions as a function of the communication length scale. Simulation-determined transition points are drawn with blue markers, and correspond to the smallest $\lambda (l_{2})$ for which a swarm, which is initially prepared in a MS, keeps all agents from reaching a distance $10\cdot l_{2}$ from the center-of-mass after an integration time of $t = 1000$. In subpanel (a), the top series represents shedding of a $k = 4$ agent, the middle to $k = 6$, and the bottom to $k = 8$. In the panel (c), we show a snapshot of the swarm for $\lambda$ just below the critical point. We can see that one agent flies away from the rest of the network (in the bottom right corner), and will continue on to infinity. In contrast, in panels (b) and (d) a single delocalized shedding transition occurs given a Watts-Strogatz network topology, where the underlying degree-distribution was sharply peaked around the average $\langle k \rangle = 10$ with a standard deviation $\sigma = 1.4$. For reference, the Watts-Strogatz model produces “small-world” networks by adding a small fraction of random short-cuts to a ring lattice^59^. As shown in Fig.4d, all agents fly away from the swarm center, independently, for $\lambda$ just below the critical point. The swarm effectively breaks up into a collection of disconnected agents, with $W_{ij} \rightarrow 0$ for all but a small fraction of interactions with $A_{ij} \ne 0$. See supplementary material for more network details, including degree-distribution plots.

### Shedding theory

Using our mean-field theory it is possible to predict the shedding transition in Fig. 4, and thus gain a more quantitative understanding. Within the mean-field, shedding corresponds to the disappearance of stable solutions to Eq. (3a) in *saddle-node* bifurcations (SNs), shown in Fig. 3 where two branches of MS radii collide—the red and blue curves. Consequently, we expect a single eigenvalue of the linearized Eq. (3a) to approach zero as $\lambda \rightarrow \lambda _{c}$. We can find a general condition to determine the critical coupling at the SN through the following. First, we compute the derivatives of Eqs. (3a) with respect to the MS radii, $\partial F_{m}/\partial r_{n}$, which constitute the elements of a Jacobian matrix, ${\underline{J}}$, where $J_{mn} \equiv \partial F_{m}/\partial r_{n}$. Then, we impose that the the largest eigenvalue of ${\underline{J}}$, denoted $\mu _{N}$, is zero:

6 $$\begin{aligned} \mu _{N}=0, \end{aligned}$$

where $\mu _{1}\le \mu _{2}\le \cdots \le \mu _{N}$ are the eigenvalues of ${\underline{J}}$. When Eq. (6) is added to Eqs.(3a), the result is $N + 1$ equations for the first shedding transition point, $\lambda _{c,1}$, and the associated MS radii.

Numerical mean-field solutions for shedding transitions in Fig. 4 are plotted with lines and show excellent agreement with simulations over a range of communication length scales, $l_{2}$. Shedding-transition simulations were performed by first integrating Eq. (2) from the initial conditions specified in the first paragraph of ‘Methods’ section for fixed parameters, starting at $\lambda = 6$. Then, $\lambda$ was reduced by 0.001 and the system was integrated for another $\Delta t = 1000$. At which point, the number of agents within a distance $10\cdot l_{2}$ from the center of mass was recorded, $\lambda$ reduced again, and the process repeated. Measured transition values corresponded to $\lambda$ at which the number of agents within the $10\cdot l_{2}$ range changed from one increment to another. This set of numerical experiments was repeated for multiple values of $l_{2}$.

In addition, the predicted unstable mode at the SN associated with the eigenvalue $\mu _{N} = 0$, i.e., ${{\mathbf{0}}} \equiv {\underline{J}}{\mathbf{v}}_{N}$, is plotted in the inlets of (a) and (b) for both examples; in particular for each agent *i*, $\text {v}_{N,\;i}$ is plotted versus its degree $k_{i}$. We can see that the non-zero components of the mode predict accurately which agents are shed in Fig. 4c, d. In the localized case, the mode in the inlet of (a) is approximately zero for all agents, except one at $k = 4$. In contrast, for the delocalized case the mode in the inlet of (b) is homogeneously distributed across all agents. We note that for predicting the second and third localized transition curves in panel (a), we simply remove nodes from the network with non-zero elements in this mode. In fact, for the all three curves in Fig.4a, there is only one such node for each transition line. Each subsequent (lower) mean-field transition line is computed from Eq. (3a) using the *residual network*, and deleting edges in $A_{ij}$ that correspond to ejected nodes in the previous shedding transition.

An interesting consequence of our SN theory is the prediction that shedding still occurs even in the limit of zero-repulsion, $l_{1} \rightarrow 0$, which one can check by looking for solutions in such cases. The implication is that shedding depends on the finite communication range, $l_{2}$, and the sparse network topology, $\langle k \rangle \ll N$. We can demonstrate this by performing an explicit calculation for random networks that are approximately degree-homogeneous, where all nodes are assumed to have the same degree, $k_{i} \approx \langle k \rangle$ $\forall i$, as in the Watts–Strogatz example. For such networks, we expect the MS radii to be equal, $r_{i} = r$ $\forall i$. Consequently, the integral in Eq. (3a) can be simply evaluated. By usefully defining a normalized radius, $a\equiv r/l_{2}$, Eq. (3a) reduces to

7 $$\begin{aligned} 0 = \frac{\alpha }{\beta \langle k \rangle l_{2}^{2} \lambda } +\;\frac{4a^{3}+6a^{2}+6a+3-3e^{2a}}{2a^{2}e^{2a}}. \end{aligned}$$

We can calculate the critical threshold, $\lambda _{c}$, by setting the derivate of the right-hand-side of Eq. (7) with respect to *a* equal to zero, as implied by Eq. (6). The result is a root equation for the critical (normalized) radius $a_{c}$. The radius is independent of all other model parameters and satisfies

8 $$\begin{aligned} 0 = 4a^{4}_{c} +4a^{3}_{c} +6a^{2}_{c} +6a_{c} +3 -3e^{2a_{c}}. \end{aligned}$$

It is easy to check that Eq. (8) has a single positive solution, $a_{c} = 1.41872271133$—quantitatively demonstrating that shedding occurs when MS radii reach the $l_{2}$ communication length scale, $a \approx 1$. Finally, by combining Eqs. (7–8), the critical coupling reduces nicely to the product of model parameters and a pure number

9 $$\begin{aligned} \lambda _{c}= \frac{\alpha }{\beta \langle k \rangle l_{2}^{2}}\;\frac{2a_{c}^{2}e^{2a_{c}}}{3e^{2a_{c}}-4a_{c}^{3}-6a_{c}^{2}-6a_{c}-3}\;\;. \end{aligned}$$

For reference, the pure number in Eq. (9) is 4.240937302. For the Watts–Strogatz network the prediction Eq. (9) is nearly indistinguishable from the more accurate calculation that employs the exact network Jacobian, Eq. (6). In fact, the two predictions agree to within $1\%$ for all $l_{2}$ when $l_{1} = 0$.

On the other hand, in degree-heterogeneous swarming networks, such as the power-law and Waxman networks, single-node shedding is the most typical, and the general SN condition Eq. (6) reduces to

10 $$\begin{aligned} \lambda _{c}=\;\alpha \bigg / \beta r_{m}^{2} \sum _{j}A_{mj} \int _{0}^{\pi } \frac{\sin {\theta _{j}d\theta _{j}}}{2}\;\frac{e^{-\frac{d_{mj}}{l_{2}}}(r_{j}\cos {\theta _{j}}-r_{m})^{2}}{d_{mj}}\;\left( \frac{l_{1}-d_{mj}}{(r_{j}\cos {\theta _{j}}-r_{m})^{2}} -\frac{l_{1}}{d_{mj}^{2}} +\frac{\left( 1-l_{1}\left/\right.d_{mj}\right) }{l_{2}} \right) , \end{aligned}$$

where the subscript *m* corresponds to the node with the minimum degree, $k_{m} = \min _{i}\{k_{i}\}$. If multiple agents have degree $k_{m}$, and are not directly connected in the network, then Eq. (10) is only satisfied for one node—the node with the largest radius. Predictions in Fig. 4a were computed from Eq.(10) in this way.

A related localized shedding case occurs in heterogeneous networks when two (or more generally, *n*) nodes with degree $k_{m}$ are directly connected. In this case, all *n* nodes are shed simultaneously, and it is straightforward to generalize Eq. (10). The calculation reduces to setting the determinant of an *n*-by-*n* Jacobian sub-matrix to zero. For example in the power-law swarming network, the first shedding transition corresponds to the loss of two directly connected $k = 10$ agents in the MS. The radii of both nodes associated with this transition are plotted in Fig. 3a.

## Discussion

In this work, we provided analytical insights into swarm cohesion under sparse interaction network constraints by adding explicit interaction graphs into a well known and general physics model for swarm pattern formation^39–41,50,54^. Using the more general networked interactions, we introduced the phenomenon of swarm shedding whereby weakly-held agents in a swarming network are ejected from collective milling states, where there is no velocity consensus and no net motion of the swarm’s center of mass. We distinguished between localized and delocalized shedding in degree-heterogeneous and homogeneous networks, respectively. In the former, one (or a small number) of agents are ejected from a mill as the coupling-strength or interaction range is reduced. In the latter, all nodes are ejected simultaneously as a swarm breaks up into effectively disconnected agents. Such transitions were accurately described in terms of saddle-node bifurcations of circular-orbit limit cycles within a mean-field approximation, and agreed well with numerical simulations. This network-based swarming theory will guide new physics-inspired swarm robotics experiments, where earlier instantiations effectively assumed all-to-all communication, and hence, may not be easily scalable to larger robotic swarms, especially in complex environments^42,46^.

Though our analysis dealt directly with self-propelled swarming networks with position-dependent, finite-range interactions, our basic approach could easily be extended to a broader range of models. An important next step would be to extend our analysis to network systems with explicit time-dependent topology, and not just position-dependent interaction weights—particularly for swarming applications in robotics. Another important extension would be to consider noise-induced shedding, since our theory implies the existence of saddle milling states through which networks are expected to break-up into smaller swarms in the presence of noise^60^. Finally, understanding the differences between shedding from milling states and shedding from flocking states in swarming networks, represents an interesting question for future comparisons. These and other implications of our shedding theory will be explored in future autonomous mobile-robot experiments.

Altogether, this work takes an important step toward further understanding the role of complex network topology in facilitating coherent motion in self-organized swarms of mobile agents, and provides insight into how such patterns can change stability through the loss of agents.

## Supplementary Information

Supplementary Information.
