# Supplementary material for: Swarm shedding in networks of self-propelled agents
Source: Sci Rep. 2021 Jun 29;11:13544. doi: 10.1038/s41598-021-92748-1 (PMC8242078; doi:10.1038/s41598-021-92748-1)
Supplement: Supplementary file 1 — Supplementary Information. [file 41598_2021_92748_MOESM1_ESM.pdf]

Appendix: Swarm shedding in networks of self-propelled agents

Jason Hindes[1], Victoria Edwards[2], George Stantchev[1], Klimka Szwaykowska Kasraie[3] and Ira B. Schwartz[1]
[1] *U.S. Naval Research Laboratory, Washington, DC 20375, USA*
[2] *University of Pennsylvania, Philadelphia, PA 19104, USA and*
[3] *Georgia Tech Research Institute, Atlanta, GA 30318, USA*

I. Network models

The purpose of showing milling and shedding results on several underlying networks was to demonstrate the robustness of our analysis to variations in a swarm's topology. The analysis took as input the backbone adjacency matrix, A_{ij}, which is user-specified and assumed arbitrary. Nevertheless, we summarize the exact parameters for network construction here for completeness. The three random networks discussed in the main text were constructed using the python module NetworkX[1].

For the Waxman geometric random graph, 300 nodes were placed uniformly at random across a 2-dimensional rectangular grid of unit length, with each pair of nodes connected with probability $p = 0.3 \cdot \exp\{-D/0.2 \cdot \sqrt{2}\}$, where D was the distance between the nodes in the grid and $\sqrt{2}$ was the maximum such distance. The Waxman construction is a common model for ad-hoc wireless networks[2]. On the other hand, for the power-law network, 300 nodes were randomly assigned a degree, k, from the probability distribution $g(k) = k^{-2.5}/\sum_{k'=10}^{300} k'^{-2.5}$. Edge "stubs" were then connected uniformly at random according to the configuration model[3]. The random power-law construction is a common model for social networks. Lastly, for the Watts-Strogatz random graph, a ring network was constructed with 100 nodes, each with exactly 10 connections. Every edge in the original network was then rewired with probability $p = 0.2$. The Watts-Strogatz construction is a common model for "small-world" networks, in both physical and social systems, which exhibit clustering and relatively short distances between every node[4].

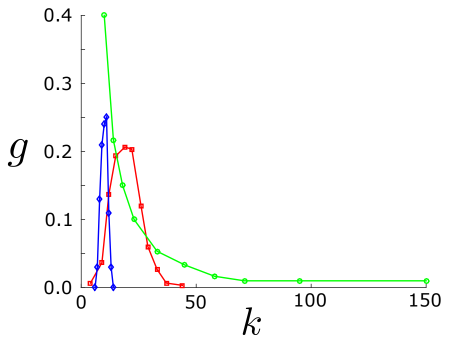

FIG. 1. Degree distributions for the example swarming networks. Plotted is the fraction of nodes with degree k: Watts-Strogatz (blue diamonds), Waxman (red squares), and power-law (green cricles) networks.

All parameters specified in the preceding paragraph can be passed as arguments to NetworkX functions, which create random realizations of each graph. In Fig.1 we plot the fraction of nodes with a given degree for each network. As mentioned in the main-text the networks can be roughly arranged in terms of the broadness of the degree distribution, or heterogeneity: Watts-Strogatz (homogeneous, blue), Waxman (somewhat heterogeneous, red), and power-law (very heterogeneous, green).

[1] A. A. Hagberg, D. A. Schult, and P. J. Swart, in *Proceedings of the 7th Python in Science Conference*, edited by G. Varoquaux, T. Vaught, and J. Millman (Pasadena, CA USA, 2008) pp. 11 – 15.

[2] B. M. Waxman, IEEE Journal on Selected Areas in Communications **6**, 1617 (1988).

[3] M. E. J. Newman, S. H. Strogatz, and D. J. Watts, Phys. Rev. E **64**, 026118 (2001).

[4] D. J. Watts and S. H. Strogatz, Nature **393**, 440 (1998).
